# A Study on Small Clinics Waste Management Practice, Rules, Staff Knowledge, and Motivating Factor in a Rapidly Urbanizing Area

**DOI:** 10.3390/ijerph16204044

**Published:** 2019-10-22

**Authors:** Bilal Ahmed Khan, Aves Ahmed Khan, Haris Ahmed, Shazia Shaheen Shaikh, Zhaiming Peng, Longsheng Cheng

**Affiliations:** 1 School of Economics and Management, Nanjing University of Science and Technology, Nanjing 210094, China; bilal2kkb@yahoo.com (B.A.K.); shaziakbshaikh@yahoo.com (S.S.S.); pz_ming@njust.edu.cn (Z.P.); 2 School of Economics and Management, Southeast University, Nanjing 211189, China; 3 Institute of Business Administration, University of Sindh, Jamshoro 76090, Pakistan

**Keywords:** healthcare waste, small clinics, exploratory factor analysis, waste generation, regulation

## Abstract

Thousands of small clinics in Pakistan are generating dispersed medical waste, unlike large hospitals, small clinic waste management is often ignored. This study was conducted on 135 small clinics in Hyderabad, Pakistan, with the aim to determine small clinics’ waste management practices in contrast to rules, level of knowledge, the environmental impact of disposal methods, and motivating factor analysis to understand the current situation from multiple perspectives. Overall, the waste generation rate was calculated to be 2.01 kg/clinic/day and the hazardous waste generation rate was 0.89 kg/clinic/day, whereas the general waste generation rate was 1.12 kg/clinic/day. The hazardous waste generation rate percentage is found to be higher than those found in large hospitals by 20%. The waste management practice among surveyed clinics was deplorable; none of the clinics were completely following hospital waste management rules of 2005 and thus the absence of proper segregation, storage, transportation, and disposal was commonly encountered during the study. Clinic staff possessed low level of knowledge and awareness, and acquired no training about waste management practice and rules, moreover, frequent employee turnover was noticed too. Additionally, two hypotheses were checked for creditability of motivating factors with an exploratory factor analysis to check their contribution to motivating clinic staff to practice sound healthcare waste management. Out of 10 indicators, nine were found in support of the hypotheses. Hence, it was discovered that active government involvement and financial support in providing training and inspecting small clinics could help in improving the condition. The findings of the present study can play a vital role in documenting evidence, and for policymakers and governments to plan solid waste management of small clinics and other healthcare facilities.

## 1. Introduction

Amongst all the substantial challenges for today’s world, healthcare waste management will make it among the top lists. Mishandling of healthcare waste threatens the health of the surrounding population and environment [1]. Previous studies have presented data that clinical waste mishandling has been causing the transmission of several fatal diseases such as hepatitis viruses B, C, and human immunodeficiency virus (HIV) [2]. Hence, it is safe to say that inappropriate healthcare waste management is a clear threat to human health.

This waste is generated in healthcare facilities during treatments, immunizations, and the diagnoses of patients. Generally, healthcare waste is divided into two categories; one is general waste, and the other is an infectious waste [3,4,5]. According to a World Health Organization (WHO) publication from 2013, waste from healthcare facilities contains 75% to 90% non-hazardous waste, whereas the rest is considered infectious [6]. Moreover, due to the increase in the use of innovative and high-tech practices and safety concerns, disposable equipment is more commonly used in developed countries, which results in generating more and more healthcare waste in comparison to developing countries [7]. Developed countries have articulated new technologies and data management systems along with adequate rules and policies for waste management, which are easily enforced as a result of access to sufficient resources [8,9,10].

Multiple studies have highlighted improper healthcare waste management practice issues in developing countries. Akter in 2000 stated that “There is no proper waste management system in place in most developing countries” [11]. In developing countries, healthcare waste management is assigned to the municipal waste handler without providing any specialized training or instruction about handling such waste differently [12]. A study conducted in the Indian region has also stated that improper healthcare waste management practice, no proper segregation, no safe collection, and transportation has been widely reported [13]. Healthcare waste from such facilities is often found to be dumped in the community bin, which should only be used for municipal waste [14]. A study from Bangladesh stated that most healthcare facilities do not have any proper healthcare waste, and only a few private hospitals are following rules to some acceptable extent. The same study also reported that people who are engaged in healthcare waste management are also found to be illegally recovering sharps, saline bags, blood bags, and other resale able items, which is later reused [15]. Many healthcare facilities use different disposal methods such as burning, dumping, burial, and reuse. Apart from these Asian countries, similar findings have been reported in other studies conducted in different resource-constrained countries such as Brazil, Jordon, Iran, and Ghana [7,16]. Previous studies have mostly focused on large healthcare facilities, and small clinics have only given a little focus. We have taken it a step further and deeply analyzed the healthcare waste management condition of such previously ignored subjects.

To some extent, large hospitals safely manage their waste; however, as most of these large hospitals are government-supported or private investors have established them as large businesses, they generate sufficient funds for managing their waste properly. However, there are hundreds of small clinics across cities which fail to follow proper waste management methods due to many different reasons [17]. These small clinics are as essential as large hospitals as they serve a very large number of population in different areas and generate a significant amount healthcare waste, in Brazil, 20% of the healthcare waste is produced by around 4000 small clinics [18], which if not managed properly, can cause a serious threat to peoples’’ health and environment. Pakistan is a rapidly growing country [19], where small clinics and their improper healthcare management is no exception. The WHO published a report in 2017, which stated that the private sector covers 60% to 70% of healthcare services in Pakistan [20]. Small clinics can be found in almost every city’s every part, as well as in small towns and villages which also have such small clinics [21]. These clinics are owned by a single doctor as a full- or part-time business. Waste generated in small clinics is almost similar to large hospital waste; it includes sharps, glass, body tissue, blood polluted cotton buds and dressings, and other kinds of infectious items. Therefore, countries across the world have made rules for healthcare waste management practices to ensure safety [22]. Implementing proper healthcare waste management practice is an unignorable need [23]. The country’s federal Ministry of Environment issued and implemented hospital waste management rules in 2005 [24]. These rules apply to all healthcare facilities, including clinics, laboratories, pharmacies, health units, dispensaries, nursing homes, maternity centers, autopsy centers, blood banks and others related to biomedical activities. Hence, it is the responsibility of all healthcare facilities including small clinics to manage their waste through proper methods, and this is necessary for the protection of human health and the environment as well. All healthcare facilities and waste management departments must follow proper rules and guidance of their country such as the hospital waste management rules of 2005 in Pakistan.

The main advantages of proper clinical waste management are connected to human health and safety. 

The present study was designed for analyzing the healthcare waste management condition in small clinics. It combined different approaches, which included waste generation data analysis, determining small clinics’ waste management practice in contrast to rules, current knowledge and awareness among staff members, and motivating factors analysis for sound waste management. Clinics from a densely populated large city in Pakistan—Hyderabad were surveyed. Several previous studies have been conducted on social, economic, and behavioral motivating reasons behind proper municipal waste management and large hospital waste management [25]. However, small clinics waste management has remained entirely untouched.

## 2. Materials and Methods

For this study, the material was collected using both primary and secondary sources. Primary sources included approaches such as one to one interviews, questionnaires, and physical waste measurement. Additionally, personal observation data was also recorded related to current waste management practice. Secondary data sources included government documents, books, previous studies, newspapers, and other internet sources. The study was carried out in four phases as follows:

Phase 1: Collection of waste generation data.
Phase 2: Determination of current healthcare waste practice in contrast with rules.
Phase 3: Assessing waste management knowledge and awareness among clinic staff.
Phase 4: Motivating factors analysis for sound waste management practice.

The study was initiated with a survey for phase 1; conducted of clinics in which quantitative data was collected from selected small clinics for an entire week in August, 2018. Collected data was comprised of a waste generation record. Waste generation data was recorded by physically measuring waste after segregation. Waste was recorded under the two main heads of hazardous waste and general waste; these two heads were further divided into sub-categories [26,27]. Medical waste was categorized into sharps, glass, plastic rubber, paper, contaminated dressings, swabs, and bandages while general waste consisted of fruit peels, cigarette filters, matchsticks, empty juice packs and bottles, paper, and polyethylene bags. The physical measurement of waste was done on a daily basis for a consecutive seven days on-site, for which the clinic’s staff was provided with a digital measuring scale. Before the measurement, the clinic’s staff was asked to participate voluntarily, and they received brief training on data collection methods.

Phase 2 was based on personal observation of the current waste management practice of small clinics; observed data was recorded on waste segregation, waste storage and transportation, and waste disposal and resale. A datasheet was designed for collecting information under specified heads. Later, collected data was matched with the country’s current rules of hospital waste management (HWM) of 2005 [24].

A qualitative survey consisted of a questionnaire (see Table S1) for individuals employed at small clinics, commonly called the compounder of the clinic was conducted for phase 3. The objective was to fully understand the level of staff knowledge about healthcare waste management.

Phase 4 was designed to analyze the key motivating factors with the help exploratory factor analysis (EFA), for which data collected through a second questionnaire was analyzed. Several previous studies have been conducted on social, economic, and behavioral motivating reasons behind proper municipal waste management. Studies on small clinics personnel are equal to none. Indicators were developed to measure positive incentives for sound small clinics waste management by using existing literature on healthcare waste management. As per the specialized nature of this study, necessary changes were made. Thus, for this survey, it was hypothesized that a small clinic’s staff decision to adopt proper waste management practices is positively associated with the perception that:

- Positive behavioral changes towards waste management can be achieved with financial support.
- Active government involvement can improve small clinic waste management conditions.

For this study, three urban Talukas of the district Hyderabad have been selected, 45 small clinics were surveyed from each Taluka for data collection, data were collected from selected small clinics for an entire week in August 2018. The survey consisted of a questionnaire based on two hypotheses mentioned above; it contained ten questions (see Table S2).

This was designed to analyze possible motivating factors which can bring positive changes in staff attitude towards healthcare waste management practice. The questionnaire was given to small clinic staff members only; no doctors were included in the survey as they do not directly handle the waste generated at their clinics. The survey was only conducted after relevant permissions were granted from the concerned owners/managers at each clinic. The analysis and findings of the quantitative data correspondingly sought to test the proposed research hypotheses. Permission was taken from the supervising professor; all the instructions and suggestions were followed during the survey. One hundred and thirty-five filled in questionnaires were received, among which three were rejected due to incomplete responses. After collecting data through the questionnaire, exploratory factor analysis was used in statistical techniques using Statistical Package for Social Sciences (SPSS) version 25.0 for testing the data.

### 2.1. Data Validity and Reliability

Prior to the distribution of the questionnaire; face and content validity were checked; an expert was contacted for assessment of the content, and research ethics were taken under consideration. Simple language format was used, and to avoid confusion, questions were kept as short as possible. To check the survey instrument reliability, the questionnaire was carefully tested for internal consistency with Cronbach’s alpha score in the SPSS statistical package version 25.0. All these steps were taken to ensure the reliability of the tools. The questionnaires were given to the staff member of 135 clinics from all three Talukas, these clinics were randomly selected, and all were within the Hyderabad district.

### 2.2. Consent

We obtained the consent for this study by attaching a consent letter with the questionnaire, which was read and explained to the responded. The consent letter was used for the purpose of introducing the research topic and research benefits; it also asks for the willingness of the responded to participate voluntarily. Furthermore, the letter explains how the participants are chosen for this study, and if they want, they can withdraw from it.

### 2.3. Ethical Consideration

In qualitative studies, it is essential to consider ethical norms, as there are direct relations of respondents personal and social life, which can affect it in many ways if not taken care carefully. Ethical consideration, as explained by Berg and Lune in 2012 for qualitative research, was followed during the survey. The point followed, known as “do not harm”, referred to the stipulation that there should be no emotional or physical damage to the subjects. The study was conducted within ethical limits, the subjects’ and institutions’ identities were not disclosed, and questionnaires did not include any health-related or personal questions. No sensitive information was leaked in any written form; subjects anonymity was ensured; no true names were mentioned for either clinics or respondents. Additionally, respondents were asked if they have any other information which they did not want to show all given details for and these were made confidential.

## 3. Results

### 3.1. Waste Generation

A consolidated waste generation rate of waste measured at clinics was calculated to be 2.01 kg/day/clinic. The medical waste generation rate of all clinics turned out to be 0.89 kg, whereas, general waste was calculated to be 1.12 kg/day/clinic. The hazardous waste generation rate was in small clinics found to be 44.18% of the total waste generation rate, which showed a significant difference between the hazardous waste rate of large hospitals [25]. Further, these clinics were grouped according to the location in the district’s taluka. Data from 135 clinics from three different talukas of district Hyderabad, namely Qasimabad, Latifabad, and Hyderabad City was collected; these talukas were abbreviated as Q, LTF, and HC, respectively. One week’s waste generation data from each taluka is given category-wise in Table 1. The waste generation rate (WGR) of each taluka was separately calculated in which WGR of LTF turned out to be the highest followed by Q and HC, respectively. Total WGR of taluka Q was 2.01kg/clinic/day, in which hazardous waste was 44.81%, and general waste was 55.19%. Similarly, for taluka LTF, the total WGR was 2.11kg/clinic/day, hazardous waste holding was 45.77% and general waste was 54.22%. HC taluka has the lowest total WGR among all three talukas, 1.91kg/clinic/day, comprised of 41.77% and 58.22% of hazardous and general waste, respectively.

### 3.2. Waste Management Practices in Hyderabad, Pakistan

#### 3.2.1. Waste Segregation

HWM rules 2005, section 16, consists of eight points about the segregation of healthcare waste management, in which it is clearly stated that hazardous waste must be separated at the generation point by the first person. However, it has been observed that waste segregation was not practiced by most of these clinics, during the data collection period staff were especially asked to segregate and measure the waste. No color coding or labeling procedure was found known to the staff members. Point 2 of section 16 states that all used supplies must be rendered as being in a non-usable condition at the point of use. Many clinics from talukas LTF and Q have been found to implement on this rule, yet many clinics in HC found to be completely ignoring this practice.

#### 3.2.2. Waste Storage and Transportation

Moreover, it has also been observed that waste was not disposed of in time, in some clinics, waste was kept for days in the same waste containers. Whereas, according to the rulebook section 19, point 4 mentions that waste must not be kept more than 20 hours. Removal of waste bags in time is an essential part of waste management, according to previous studies [2]. Waste dispersion near the waste containers was also seen in many clinics where waste was kept for a long time.

Section 18 of HWM rules 2005 containing five subsections about waste transportation rules. Many previous studies on large hospitals have found that waste transportation has mixed practice scenarios; some hospitals were found to follow the rules partially, whereas some are not [25]. Unlike large hospitals, these small clinics waste transportation facilities were equal to zero; there were no transportation arrangements found for any of these clinics.

#### 3.2.3. Waste Disposal and Resale

The HWM rule book, section 20, with 11 sub-sections gives details about the waste disposal process. Many previous studies have also outlined similar and more advanced waste disposal methods for hazardous waste—namely incineration, steam sterilization, microwave sanitation, dry and chemical disinfection, and many more [28]; however, most small clinics were found to be ignoring them. It has been observed, that not all small clinics practiced the same waste management methods, as some directly threw their waste in the nearest municipal waste pile, whereas a large number of clinics practiced unsegregated open burning near the clinic. It has also been observed that some of the waste, such as plastic, glass, and sharps were also found to be sold to individual waste buyers or taken by scavengers. It has been reported in previous studies that medical waste is illegally recycled in some of the developing countries [29]. Medical waste is used to make drinking straws, lollipop sticks, and toys; such practice is undoubtedly a direct threat to the health of children [30]. Surveyed clinics were no exception and all these unsafe waste practice elements were observed during the study.

### 3.3. Knowledge and Awareness Among Clinic Staff

A 10-question based questionnaire was used to gather information about knowledge and awareness of clinic staff about clinical waste management. Table 2 shows all the questions and their corresponding correct response percentages. The first question inquired if the staff were aware of the current rules of healthcare waste management in the country, only 19% of the surveyed staff members were able to answer this question correctly. The country is currently following the HWM rules of 2005, and all healthcare facilities are bound to implement these rules. However, implementation is equal to none when it comes to small clinics. This lack of implementation is due to a very small portion of the hired staff members being aware of these rules.

The second question was aimed to know if staff were aware of the importance of incinerating medical waste, which resulted in only 33% correct answers. Many of the staff members were found to be aware that infectious material needs to be incinerated as it can cause different viruses to spread. However, a much larger number of the clinic staff did not have any information about the incineration of medical waste. Additionally, there were no incineration plants available for such waste from small clinics; the only one incineration plant in working condition was used for large hospitals of the city.

Question number three aimed to collect data about awareness of color-coded waste bags. According to healthcare waste management policies, waste should be gathered in predefined color-coded waste bags to avoid mishandling and injuries. Such color-coding can help the waste handler to perform their job more efficiently and reduces the chances of error. Staff members from surveyed clinics were mostly unaware of this, and only 27% of them were able to answer correctly. The possible reason behind this lack of awareness can be the unavailability of color-coded waste bags as, during our survey, none of the clinics were found to have these specialized bags to store waste. Therefore, naturally, staff members may not have paid any attention to this piece of information.

Question four was designed to find out if clinic staff were aware of the importance of disposing of the waste within a safe time zone; this is very important for the staff members to be aware of as keeping waste for a long period of time can cause many risks. Waste containers must be emptied and disinfected before reusing them and this act mitigates the risk of waste dispersion and degradation. According to collected data, only 39% of the staff members were aware of the importance of waste disposal within a safe time, which is considered a very low level of awareness.

The highest correct answer percentage was received for questions six, eight, and five with 89%, 79%, and 69%, respectively, none of the other questions received a higher or equal percentage of the correct answer than these three. Question five which stands third among the top three was aimed to check whether staff members were aware of the safety equipment required for handling waste, these equipment were one of the key steps to avoid injuries and infections, and the utmost beneficiary of the use of the proper equipment was none other the staff members themselves. Whereas question eight, with the second-highest percentage, was designed to find out if the clinic staff were aware that some of the used medical supplies needed to be destroyed before disposal, as such items include syringes and other sharps. Many studies and news reports have brought up the issue of reusing infectious items at many medical facilities in underdeveloped and developing countries. There are strict laws in developed countries, and high implementation is recorded, which attempt to prevent the spread of disease in the region. Question number six topped the list of correct answers and this question aimed to find out which government department is responsible for healthcare waste, which had a very obvious answer as the highly congested urban areas of the country were facing continuous problems of municipal waste and gutter blockages and everyone was found to be aware of the department responsible for this.

The lowest rate of awareness was found for the waste data records in clinics; only 9% of the staff were aware that they are supposed to maintain waste generation data. During the survey, none of the clinics were found to have any kind of waste generation records, this is an essential part of waste management, especially for waste as sensitive as medical waste, which directly affects human health. However, a shallow level of awareness among clinics staff has wholly ignored this step. The last question gathered the second-lowest percentage of correct answers with only 17%; this question was about biohazard signs on waste containers. A minimal number of clinics staff were aware of the signs on the container; however, none were found to be practicing this as there were no safe waste containers that were provided inside the clinics. Most of the clinics did not spend any amount on managing their waste as their clinics did not generate a sufficient amount.

### 3.4. Factor Analyses and Hypotheses Validation

Responses of 10 indicators on a five-point Likert scale were measured, and an observation to variables ratio 5:1 was achieved. The scale development phase was completed based on a previous study [31]. Furthermore, this scale was created to get more précised measurements, better chances of detecting changes, and to have a better explanation about the point of view [32]. Table 3 presents the descriptive analysis in which mean, standard deviation, and number are given in each corresponding variable column.

Consistent, reproducible, and stable measurement is necessary for reliable results [33]. Reliability is a very significant factor for knowing the quality of measurement instrument, as it assists in identifying the impact of inconsistencies on the measurement results. Bryman and Cramer stated that if there are multiple measurement items within constructs, internal reliability is primarily significant [34]. This research consisted of the measurement of multiple items; therefore, the reliability of the measurement items was assessed with the consistency of the answer from respondents [35]. To measure internal consistency, Cronbach’s alpha reliability coefficients were used, and less than 0.6 reliability coefficients were considered to be poor, acceptable were between 0.6 and 0.7, and all greater than 0.8 were considered as good [33]. According to Nunally, all Cronbach’s alpha reliability coefficients, which are 0.7 or above can be considered adequate [35]. Whereas, Hair et al. stated that all 0.7 or above present adequate internal consistency [36]. Based on these suggestions a minimum cutoff value of 0.7 for Cronbach ’s alpha reliability coefficients was used for determining the reliability of individual measures to find out the total reliability of each latent constructs used in this study.

The data was converted into an interpretable format [37], and key characteristics of the data are presented in descriptive statistics, including alpha means and standard deviations of study variables. All latent construct items were rated on the five-point Likert scale, where five represents strongly agree, and one represents strongly disagree with each question in the questionnaire survey.

The primary purpose of cross-loading is to check all the items either loaded in their construct with high scores or not; this researcher has suggested using a new developmental scale, where 0.50 or higher should keep in the measurement model. Hulland (1999), suggested that items which were loaded below 0.5 should be removed from the model. EFA was used for analyzing the results of the study to confirm model tangibility. A variable correlation matrix was used to explore the multicollinearity of data. Multicollinearity was not an issue since all the correlations were greater than 0.05, and the determinant of the matrix was 0.01 (>0.00001). The Kaiser–Meyer–Olkin (KMO) test was used to check the sample adequacy shown in Table 4.

The KMO test value was 0.595, which was higher than the least required value of 0.5. A lower than 0.001 *p*-value was detected in Bartlett’s test. We rejected the null hypothesis for being a correlation and the identity matrix. Using principal component analysis (PCA), two factors with eigenvalues greater than one were extracted, and these factors represented 73% model variance. With minimum cross-loadings, the factors were rotated by varimax for getting a simple structure [38]. Due to the small size of the sample, only factors having higher than 0.5 values were retained [39]. For the cross-loading items, we have used 0.2 as a cutoff value [40], the questionnaire used for this survey had 10 questions based on two hypotheses named “active government” and “financial support”. Questions one, two, four, five and six were formed to support the “active government” hypothesis, whereas questions three, seven, eight, nine and 10 were for the second hypothesis namely “financial support”. A five option Likert scale questionnaire was offered to 135 staff members of small clinics in the survey, three were rejected due to incomplete answers, and finally, a total of 132 complete responses were obtained. All the obtained responses were tested in EFA using SPSS, and 25 outputs are shown in Table 5. It is to be noted that indicators one, two, four and six fall under the factor of the active government. Similarly, indicator three, seven, eight, nine and 10 under financial support. The survey results of EFA are in favor of both hypotheses; however, indicator five was the only one indicator which showed negative factor loading. All the selected indicators have been highlighted with bold fonts in Table 5.

## 4. Discussion

This study summarizes that the waste management practice of small clinics needs urgent attention from government departments and local people. Small clinics have been analyzed from multiple perspectives to understand the current situation, which can help in solving issues related to that. Small clinics are generating healthcare waste similar to the hospital; however, the hazardous waste generation rate contained 44.18% of the total waste generation rate, which is almost 20% higher than the hazardous waste rate of large hospitals [25]. According to WHO, waste generated at a large hospital in the developing country has 10% to 25% of hazardous waste in it [1]. However small clinic waste contained more hazardous waste than that of the large hospital. One of the main reasons behind this higher hazardous waste ratio is due to the small quantity of general waste found at clinics. Whereas, in large hospitals, patients stay for a longer time period, having meals and using other daily necessities. Moreover, the patient’s family, friends, and relatives also visit—and these people ultimately ends up generating more general waste inside the hospital premises. Whereas small clinics are only visited for a short period to time, and much less general waste is generated which makes the hazardous waste portion larger. During the study it has been frequently observed that these clinics do not follow the 2005 rules of hospital waste management which are currently regulated in the country. The most frequent abuse of HWM rules 2005 was observed from sections 16, 19 and 20; these sections specifically focus on segregation, storage, and transportation and disposal of healthcare waste. However, violation of these rules is also due the ambiguities in rule formation, as the waste management condition was found worse in these small clinics in comparison to those found in large hospitals in developing countries, where poor waste management was recorded by previous studies [41,42,43]. These rules seem fit for large size hospitals, but small clinics fail to meet the criteria; therefore, it is necessary to modify these rules so that they can thoroughly apply for every kind of healthcare facility. Pakistan HWM rules need some updates according to today’s situation as these rules are more than a decade old and in the past few years technology, medical science, and populations have changed dramatically. Smaller healthcare institutes such as small clinics were often ignored while making policies due to their rare presence in the past, however, now they must be monitored by concerned departments. Policies to help in waste reduction and segregation along with inexpensive waste disposal methods should be made for small clinics in resource constrained settings. Developed countries are successful in managing their waste due to available resources for expensive treatments, whereas developing countries are unable to practice those treatments due to poor economic conditions. The results from the survey conducted to investigate the knowledge and awareness among staff members were further compared with the results of a previous study. To find out similarities in results of a study conducted on large hospitals, including government, charitable, and private hospitals in Pakistan in another economically better province are compared. It was found that the hiring process of hospitals is much better as the candidate has to go through interviews and needs to be qualified for the intended job. Hospital employees are hired for the long term, and employee turnover is lower, whereas recruitment in small clinics is more frequent as many of the staff only work for a very short time and switch to other jobs or find a better carrier opportunity. Therefore, the level of knowledge observed in clinic staff members is found to be much lower than the hospital staff members. The compared study has presented data of 114 respondents from hospitals including nurses and sanitary workers, it has been reported that these staff members are receiving regular training during their job in regard of HWM, whereas no clinics staff member has reported about any kind of training from the Government or from the clinic itself. In large hospitals, waste management is mostly carried by sanitary staff members; nurses only take part in few steps such as direct segregation of medical and general waste at the time of waste generation. Whereas, small clinics staff members are solely responsible for handling waste on all stages inside the clinic’s premises. The same study has also shown the responses of staff from Government hospitals where it was stated that 100% of nurses had received training about hospital waste management, and they were able to answer about 70% of the questions regarding HWM [44]. Sanitary staff members of the hospitals are also regularly receiving training about their job responsibilities, and about 50% of corrects answers were recorded from them. These sanitary staff members are mostly uneducated, and prior to starting that job, their knowledge level was equal to zero about hospitals waste management and how important and sensitive this matter is for their health and health of others around them. On the other hand, clinics staff members are comparatively educated, but no training has ever been provided to them. That may have caused this low level of knowledge about medical waste management. Awareness among clinics staff is so poor that most of them are not even aware of legislation and cannot fulfill their job responsibilities. It is highly suggested in many studies, over and over again, that providing training to staff members who are directly involved in handling waste at all medical facilities is essential; it can help in significantly improving overall healthcare waste management conditions. Training sessions for small clinics staff can help significantly in educating waste handlers to understand the seriousness of their jobs.

In order to find out the motivating factor, which may help in improving the overall conditions of small clinics waste management, factor analysis is done with the help of exploratory factor analysis. According to the results of this study, waste management practice in small clinics can be improved if the Government takes some initiative, as it is observed that if government officials regularly visit healthcare facilities for inspection, the demand of segregation from the Government, and providing training to staff members can improve the situation of healthcare waste management. It can be seen in Table 5, that variables related to above mention suggestion has >0.8 value, which indicated that taking such steps can bring positive changes. Along with the Government’s initiatives, financial assistance can also help improving condition another variable in Table 5 has also shown higher than 0.8 value, which is related to the availability of required equipment such as proper waste containers and others. Results shown in this study are somewhat similar to previous studies conducted on large hospital for motivating factors, due to the different size and operating methods motivating factors from large hospitals were reputation, liability, and financial burden whereas small clinics situation did not fit in for such factors. These small clinics are usually very low profiled and only serve their neighboring areas; therefore, reputation cannot be taken as an essential factor. Patients from neighboring streets do not have other choices as there is mostly one clinic in that area. Liability from government departments can cause hospital management some serious problems whereas these small clinics are often ignored by government departments, and they do not feel accountable for any kind of liability. The financial burden was the only similar factor between large hospitals and small clinics, as it is directly related to their revenue.

Studies such as this one are not merely enough to understand the overall condition. There is a critical need for research: technologically and strategically to control the continuously worsening situation of healthcare waste around the world, especially in developing countries. Cost-effective but efficient waste management systems should be designed for resource-constrained countries to manage their healthcare waste: segregated storage, safe transportation, and proper disposal. New specially designed technologies and management systems can help in minimizing cost and risk. With ever-growing urbanization and limited resources, Asian developing countries should be given high privileges to overcome healthcare waste-related issues. Governments of developing countries such as Pakistan, India, Bangladesh, Nepal and many others like these must take a step forward to solve issues related to healthcare waste in their countries.

## 5. Conclusions

Small clinic’s waste generation rate was found to be different in all three talukas of Hyderabad district overall WGR was calculated to be 2.01 kg/clinic/day, and hazardous WGR 0.89 kg/clinic/day, whereas general WGR was 1.12 kg/clinic/day. The hazardous WGR percentage was found to be higher than those found in large hospitals by 20%. This difference was due to the more extended stay of patients in a large hospital which causes more general waste. Overall, waste management practice among surveyed clinics was deplorable; no clinics were found to completely meet the standard criteria of waste management practice. Moreover, none of the clinics was following HWM rules 2005; thus, the absence of proper segregation, storage, transportation, and disposal was commonly encountered during the study.

Additionally, a shallow level of knowledge and awareness is found among clinics staff members about waste management practice and rules. Such poor waste management practice among these small clinics is mainly due to ignorance of government institutes. Moreover, HWM 2005 rules are also found to be outdated and lacks in matching the requirements of current situation. It also fails to cover small clinics of the country and mainly focused on large hospitals only. Definition section 2, point F of HWM 2005 states that these rules apply to small clinics as well; however, it has been observed most of the section does not fit on small clinics condition, and there is a need for improvement.

To find out the factor which can motivate small clinics waste management stakeholders to practice safe waste management practice. There were two hypotheses to check the creditability of the motivating factors. Nine of the ten indicators resulted in supporting the hypothesis, and their factors loading turned out to be higher than 0.5 and all nine indicators loaded in the corresponding factors. The factor of active government involvement loaded all five indicators; however, indicator five failed to achieve an acceptable factor loading figure. The other five indicators were perfectly loaded in the factor of financial support. These goals can be achieved by implementing standard protocols of healthcare waste management practice whereas, the Government needs to make some required changes and updates in rules and ensure their implementation.

The investigators should do more detailed research on the waste management practice of small-scale health facilities in different regions where HWM needs much more attention than others. Studies on the life cycle assessment of healthcare waste of small clinics should also be conducted—seasonal variation, carbon footprint, carbon credit can also be taken under consideration. A feasible solution must be discovered for all kinds of healthcare facilities, including small clinics, laboratories and so on for HWM. More in-depth studies should be conducted to find gaps in currently applied rules, and regulations which no longer be followed in current circumstances of other small facilities as most policies and regulation are specifically designed for large hospitals only. Developing countries fail to implement many policies due to the scarcity of resources. New waste treatment, which is suitable for small amounts of healthcare waste, should be introduced which are cost-effective and can be applicable in resource-constrained countries.

## Figures and Tables

**Table 1:** Waste generation data of small clinics (all units kg).

Taluka Name	Waste Type		Plastic	Paper	Glass	Textiles	Rubber	Pathological	Others	Total
Hyderabad City	Medical	Infectious	18.13	19.18	19.138	17.717	149.653	12.88		236.698
		Sharps	15.267							15.267
	General		104.91	92.414	21.539				132.272	351.141
	Total		138.31	111.594	40.677	17.717	149.653	12.88	132.272	603.106
Latifabad	Medical	Infectious	21.623	23.373	23.632	21.469	179.858	15.806		285.761
		Sharps	18.62							18.62
	General		109.28	88.487	19.306				143.437	360.514
	Total		149.52	111.86	42.938	21.469	179.858	15.806	143.437	664.895
Qasimabad	Medical	Infectious	21.07	22.645	22.036	23.114	161.147	16.324		266.336
		Sharps	18.564							18.564
	General		108.05	83.895	18.795				140.091	350.833
	Total		147.68	106.54	40.831	23.114	161.147	16.324	140.091	635.733

**Table 2:** Results of the questionnaire survey.

S.No	Questions	Correct Answers %
1	Currently, which waste management rules are being followed?	19%
2	Incineration is necessary for ________________?	33%
3	The yellow color bag is used for medical waste?	27%
4	Waste must be disposed within ______________ after generation.	39%
5	The waste handler must have the following equipment.	69%
6	In Pakistan, which government department is responsible for regulating healthcare waste?	89%
7	Where should the hazardous and non-hazardous wastes be separated?	47%
8	Which of the following equipment should be destroyed before throwing in a waste container? (check all that apply)	79%
9	When should you record the waste quantities at your clinic?	9%
10	Which of the following should have a bio-hazard sign printed or marked on it? (mark all that apply)	17%

**Table 3:** Descriptive Statistics.

S.no	Indicators	Mean	Std. Deviation	N
1	If an official person visits and tells to segregate waste, we will follow.	2.64	1.458	132
2	If government department demands segregation, we will do it.	2.66	1.222	132
3	If the government provides us some training about waste management, it can improve the condition.	3.23	1.216	132
4	If the government makes special rules for small clinics waste management, we will follow.	3.82	1.118	132
5	If special collection vehicles come for collection, we will not throw waste in municipal waste.	3.53	1.073	132
6	If the government provides, some subsidy clinic’s waste can be well managed.	2.80	1.047	132
7	If waste segregation can bring some financial benefits for us, we will do it.	2.28	1.051	132
8	If we have the proper equipment for waste segregation, we will practice.	2.16	0.818	132
9	If I am paid extra money for waste management, I will take responsibility.	2.27	1.056	132
10	If we have proper waste containers, we can practice segregation.	2.14	0.711	132

**Table 4:** Kaiser–Meyer–Olkin (KMO) statistics and Bartlett’s test of sphericity.

Kaiser–Meyer–Olkin (KMO) Statistics and Bartlett’s Test of Sphericity		
Kaiser–Meyer–Olkin Measure of Sampling Adequacy		0.595
Bartlett’s Test of Sphericity	Approx. Chi-Square	1209.784
	Df	45
	Sig.	0.000

**Table 5:** Rotated component matrix—exploratory factor analysis.

S.no	Indicators	Components	
		1	2
1	If an official person visits and tells to segregate waste, we will follow.	**0.823**	
2	If the government department demands segregation, we will do it.	**0.859**	
3	If we have proper waste containers, we can practice segregation.		**0.892**
4	If the government provides us some training about waste management, it can improve the condition.	**0.805**	
5	If the government makes special rules for small clinics waste management, we will follow.	−0.864	
6	If special collection vehicles come for collection, we will not throw waste in municipal waste.	**0.765**	0.413
7	If the government provides, some subsidy clinic’s waste can be well managed.		**0.797**
8	If waste segregation can bring some financial benefits for us, we will do it.		**0.682**
9	If we have the proper equipment for waste segregation, we will practice.		**0.770**
10	If I am paid extra money for waste management, I will take responsibility.		**0.782**

## Supplementary Materials

The following are available online at https://www.mdpi.com/1660-4601/16/20/4044/s1, Table S1: questionnaire to assess knowledge about clinical waste management among small clinic staff, Table S2: questionnaire for assessing motivation for the adoption of sound healthcare waste management practices in Hyderabad.
