# Supplementary material for: A Study on Small Clinics Waste Management Practice, Rules, Staff Knowledge, and Motivating Factor in a Rapidly Urbanizing Area
Source: Int J Environ Res Public Health. 2019 Oct 22;16(20):4044. doi: 10.3390/ijerph16204044 (PMC6843947; doi:10.3390/ijerph16204044)
Supplement: Supplementary file 1 [file ijerph-16-04044-s001.zip › Supp 1 Questionnaire to assess knowledge about clinical waste management among small clinic staff.docx]

# Questionnaire to assess knowledge about clinical waste management among small clinic staff

Name________________ Clinic Name _________________ Clinic Address____________

Experience: Less than a year More than a year

Questions:

1. Currently, which waste management rules are being followed?

Healthcare Waste Management Rules, 2005,

Healthcare Waste Management Rules, 2010

Healthcare Waste Management Rules, 2015

2. Incineration is necessary for ________________?

General waste Medical waste All kinds of waste

3. Yellow color bag uses for medical waste?

Ture False

4. Waste must be disposed within ______________ after generation.

20 hours Two days One Week

5. A waste handler must have the following equipment.

A. Gloves

B. Mask

C. Helmet

D. Safety shoes

6. In Pakistan which government agency is responsible for regulating healthcare waste wastes? (Name)______________________________________ Don’t Know

7. Where should the hazardous and non-hazardous wastes be separated?

a. at the clinic b. Outside in open air

c. at collection d. Should not be separated

8. Which of the following equipment should be destroyed before throwing in a waste container? (check all that apply)

a. Syringes b. Plastic bottles

b. Plastic drips d. Infusion bags

9. When should you record the waste quantities at your clinic?

a. Daily b. Twice a week

c. Weekly d. Never

10. Which of the following should have a bio-hazard sign printed or marked on it? (mark all that apply)

a. Risk waste bags b. Sharp boxes

c. Store Room d. Radioactive waste container
