# Supplementary material for: A Study on Small Clinics Waste Management Practice, Rules, Staff Knowledge, and Motivating Factor in a Rapidly Urbanizing Area
Source: Int J Environ Res Public Health. 2019 Oct 22;16(20):4044. doi: 10.3390/ijerph16204044 (PMC6843947; doi:10.3390/ijerph16204044)
Supplement: Supplementary file 1 [file ijerph-16-04044-s001.zip › Supp 2 Questionnaire for Assessing Motivation for the Adoption of Sound Healthcare Waste Management Practices in Hyderabad.docx]

Questionnaire for Assessing Motivation for the Adoption of Sound Healthcare Waste Management Practices in Hyderabad.

| **ITEMS** | **Strongly Disagree** | **Disagree** | **Neutral** | **Agree** | **Strongly Agree** |
| --- | --- | --- | --- | --- | --- |

| **Financial Indicators** |  |  |  |  |  | |
| --- | --- | --- | --- | --- | --- | --- |
| If the government provides, some subsidy clinic’s waste can be well managed. |  |  |  |  |  | |
| If waste segregation can bring some financial benefits for us, we will do it. |  |  |  |  |  | |
| If we have the proper equipment for waste segregation, we will practice. |  |  |  |  |  | |
| If I am paid extra money for waste management, I will take responsibility. |  |  |  |  |  | |
| If we have proper waste containers, we can practice segregation. |  |  |  |  |  | |
| **Government Involvement indicators** |  |  |  |  |  | |
| If an official person visits and tells to segregate waste, we will follow. |  |  |  |  |  | |
| If government department demands segregation, we will do it. |  |  |  |  |  | |
| If the government provides us some training about waste management, it can improve the condition. |  |  |  |  |  | |
| If special collection vehicles come for collection, we will not throw waste in municipal waste. |  |  |  |  |  | |
| If the government makes special rules for small clinics waste management, we will follow. |  |  |  |  |  |  |
